# 

**Published:** 1886-07

**Authors:** 


### INDEX—VOL. II.

A Doctor's experience in 3 Continents.................. 34
A Doctor's experience with the Law.......... ........ 34
Absorption of Cataract................................ 181
Abortion, from the Standpoint of Personal Experience.... 221
Abortion, following removal of Uterine polypus........... 363
Alas, Poor Yorick..................................... 168
American Medical Association.......................... 374
Address of President T. S. M. A........................ 70
American Medical Association, Notes from June Meeting.. 509
Analysis of the Urine.................... .... .... 36
An American Contribution to Bacteriology............... 74
A notable Address indeed.............................. 115
A dangerous operation................. ............ 121
Antiseptic, the best.................................. 352
A Prophesy....... ................................ 164
A Medical College in Texas............................ 349
A disclaimer.......................................... 392
Antipyrine, Therapeutic effects of..................... 371
Barometer, Low, in relation to non contagious Diseases... 264
Burt, Dr. W. J........................... ........ 23
Burroughs, Dr. S. R.... ............................ 499
Baking Powders and the Public Health .................. 287
Banquet or no Banquet................................. 388
Banquet, no........................................... 422
Biography, Contemporary Physicians of Texas........... 209
Books and Phamphlets rec'd............................ 351
Blood and Nutrition, Diseases of....................... 350
Best Antiseptic, the.................................. 352
Board of Censors of Johnson Co., Report of............. 375
Bell County Medical Association........................ 375
Colpo-myotomy, a case of.... ......................... 59
Craniotomy: Physician threatened with indictment....... 141
Cataract, Absorption of............................... 181

Compound comminuted, Fracture of Os Frontis.......... 189
Cervical Polypus, Abortion following removal of.......... 363
Criticism, that.......................................... 320
Curious Sequellæ in Rheumatism........................ 405
Central Texas Medical Association......................154 275
Collective Investigation of Disease...................... 121
Capital Punishment by Electricity...................... 431
Common Carotid, Ligature of, after Abscess............ 515
Compend of Pharmacy, A................................ 85
Courier Review Call Book................................ 293
Chemical Lecture Notes................................ 397
Civil Service Reform in Medicine, Do We Need A........ 25
Colored Physicians vs. Negro Doctors.................... 30
Carbolic Acid, Some New Uses for...................... 282
Coming Meeting of the T. S. M. A., The................ 332
Dr. McLaughlin's Microbic Theory of Dengue........... 232
Dengue, Dr. McLauglin's Microbic Theory of............ 232
Death of Dr. W. S. Rogers.............................. 508
Diseases, non-contagious in relation to low barometer..... 264
Diseases, Collective Investigation of.................... 121
Darwinian Theory vs. Special Creation.................. 146
Dislocation of Occipital Bone........................... 196
Disclaimer, A............................................ 392
Do we need a Civil Service Reform?................... 25
Dangerous Operation, A................................ 121
Dr. Osborn's Paper...................................... 285
Diseases of the Lung and Pleura........................ 351
Diseases of the Blood, and Nutrition.................... 352
Diseases of the Spinal Cord............................ 83
Diphtheria, Supplementary Notes on.................... 121
Dr. Rutherford........................................... 379
Dr. James H. Haley...................................... 380
Dr. W. J. Burt........................................... 23
Dr. H. W. Moore........................................ 170
Dr. Albert Welch........................................ 173
Exploratory Laparotomy................................ 402
Electricity, Methods in.................................. 506
Eclampsia, Puerperal Veratrum in...................... 228
Enucleation of Fibroid after Laparotomy, 516........... 616
Editorial Notes..................................37, 74, 125
East Line Medical Association, Action of............... 193
East Texas Medical Association..................510, 241, 279
Electricity in Placenta Prævia.......................... 312
" Capital Punishment by......................... 431
Fracture of Fibia, non-union of. Operation for........... 1
" Gunshot, of Spinal Column...................... 92

Forceps in Labor. The .... 189, 412
Fibro-Myoma, Interstitial, complicating Delivery .... 447
Fibroid, Enucleation of, after Laparotomy .... 516
Gaillard, Prof. Reminiscence of .... 9
Gunshot-Fracture of Spinal Column .... 92
" Wound of Intestines. Ex-section of portion of Intestine .... 401
Gaseous Enemata in Pulmonary Phthisis .... 503
Grateful Acknowledgments .... 501
Galveston Medical Club. Action of .... 278
Gaseous Enemata for Phthisis .... 458
Hysterotomy .... 2
Hospital Practice .... 110
Houston County Medical Society .... 513
Hour Glass Contraction, after 3rd Stage of Labor .... 144
Hydrocephalus Complicating Breech presentation .... 226
Hysteria in a young lady .... 360
Hernia, incarcerated Scrotal .... 198
He put his Foot in it .... 151
Hand-book, of General Therapeutics .... 289
Healing of Arteries after Ligature .... 292
Hand-book, Physicians for 1887 .... 294
Habitual Constipation .... 336
Haley, Dr. James H .... 380
Health Officer. The New State .... 298
Iodoform, a method of rendering inodorous .... 109
Interstitial Uterine Fibroma, Complicating delivery .... 447
Impetigo, Contagioso .... 47
Intestines, Suture of after Laparotomy .... 462
" G. S. Wound of .... 401
" Resection of after G. S. Wound of .... 401
" Exsection of a portion of .... 462
Inertia Uteri, a case of .... 63
In the interest of Peace .... 206
Insanity and its treatment .... 85
"International" County Medical Association .... 70
International Medical Congress, The ninth .... 200 112
Incarcerate Scrotal Hernia .... 198
Johnson County, Report of Board of Censor of .... 373
Jefferson Medical College .... 112
Journal Comparative Medicine and Surgery .... 291
Jury trials of Pauper Insane .... 159
Index Catalogue of Surgeon General's Office .... 292
Ligation of Brachial Artery .... 61
Laparatomy, Exploratory .... 402
" for G. S. Wound of intestines .... 401
" and Intestinal Suture .... 462

Lawson Tait's wonderful success in Ovariotomy .......... 27
Lanolin and Ichthyol.................................... 153
Ligature of Common Carotid after Abscess.............. 515
Laparotomy, Enucleation of Fibroid after................ 516
Malarial Paraplegia........................................ 110
" Hæmæturia........................................ 182
Medical Expert, The experience of a...................... 138
Microbic Theory of Dengue, Dr. McLaughlin's............ 232
Methods in Electricity .................................... 504
Medical News and Miscellany 174, 211, 259, 294, 340, 393, 429, 513
Medical College question, The........................... 389
Medical Department University of Texas................. 383
Medical Supplies, Relation of Physicians to their......... 386
Mineral Waters in Malarial Disease...................... 284
Misguided Members and The Presidents Address........ 18
Medical Legislation....................................... 11
Medico-Chirurgical College.............................. 112
Morphia, Poisoning by.................................... 274
Medical History and Biography.......................... 369
Mind your Eyes............................................ 31
Methods of Bacterial Investigation....................... 35
Manual of Practical Therapeutics........................ 84
Medical and Surgical Directory of the United States...... 86
Manual of *Diatetics* Fothergill's.......................... 293
Moore, Dr. H. W. Death of................................ 170
Meeting of the T. S. M. Association, The Coming ....... 332
Niguas, a parasite common to Mexico .................... 230
Non-Contagious Diseases in relation to low barometer.... 264
Notes on Diphtheria....................................... 121
Notes from June Meeting American Medical Association.. 509
North Texas Medical Association......................... 13
Ninth International Medical Congress..................... 200
New Way to take the Temperature, A...................... 150
New Method in Incarcerated Hernia, A................... 198
Notable Address indeed, A............................... 115
No Banquet............................................... 422
New Administration The, and State Health Officer....... 368
Obstetrics, A Case in..................................... 5
" A Difficult Case in.......................... 357
Opium Poisoning, Strychnine hypodermically in.......... 182
Oxygen in Therapeutics................................... 369
Osteo-Sarcoma, below Trochanter Major.................. 309
Obstetrics Among the Eskimo............................ 305
Placenta Prævia.........................................
Paraplegia, Malarial...................................... 110
Peri-uterine Cellulitis Simulating Pregnancy............. 134

Puerperal Elampsia.................................. 228
Placental Presentation.................................. 273
Phthisis Palmonalis gaseous enemata in.. ............... 503
Publishing Committee, What are the duties of........... 423
Phthisis, gaseous enemata for.......................... 458
Presidential Addresses and Medical Societies............ 68
Poisoning by Morphia successfully treated by Strychnine hypodermically .................................. 274
Presidents Adresses, Misguided members and............ 18
Principles and Practice of Surgery...................... 33
Prize Essay, That........................................ 242
Pharmacopœia, of the U. S. A........................... 289
Physicians visiting List, Blakistons..................... 293
Physicians Hand Book for '87........................... 293
Physicians Mutual Benefit Association.... .............. 123
Proceedings 19th Session Texas State Medical Association ..........................................474, 16, 155
Placenta Prævia, Electricity in......................... 312
Poisoning by Phytolacca................................. 319
Phytolacca poisoning by................................. 319
Paralysis, Central Bulbous and Spinal.................. 350
Programme Texas State Medical Association............ 417
Reminiscences of Prof. Gaillard.. ..................... 9
Rogers, Dr. W. S., Death of............................ 508
Resolutions by Galveston Medical Club on Death of Dr. W. S. Rogers.................... ..................... 508
Rockwall and S. E. Collin Co. Medical Society.......... 15
Rheumatism, curious sequellae in....................... 405
Quinine, Rare effects of on the skin ................... 195
Rough Experience with the Law, A Doctors............. 413
Rutherford, Dr. R....................................... 379
Requisites of a Gynæcologist............................ 208
Religion in Medical addresses.......................... 67
Rare Effects of Quinine on the Skin..................... 195
Rupture of Urethra...................................... 316
Report Board of Censors Johns on Co........... ....... 375
Relation of the Physician to his Medical Supplies........ 386
Reference Hand Book, Medical Sciences................. 288
Small Doses which are efficient......................... 152
Students Manual Venereal Disease......................
Strychnine-Hypodermically, for Opium Poisoning........ 182
Sweet Malaria........................................... 71
Spinal Column, Gun Shot Wound of..................... 92
Stricture of the Urethra, congenital..................... 355
Some New Uses for Carbolic Acid....................... 282
Synopsis of Proceedings Texas State Medical Association.. 474
State Health Officer of Texas...................... ... 379

Texas State Medical Association, Proceedings 19th meeting472 121
" " " " " ............417 372
Treatise on Practice of Medicine.......................... 357
Texas State Medical Association: Address to Members.... 511
Tait's (Lawson) Wonderful Success in Laparotomy....... 27
The Good of the Cause....................................... 117
Tait's Operation in Texas..................166, 191, 192, 103, 404
The Surgeon's Relation to Homicides before the Courts.. 203
That Prize Essay............................................. 242
Tibia, fracture of, non-union of, operation for............ 1
Transactions Texas State Medical Association........... 237
Travis County Medical Association...................... 15 418
That Criticism............ .. ........................... 320
Therapeutic Properties of Antipyrine...................... 371
Texas Surgery................................................ 394
The President Elect Texas State Medical Association..... 500
The Recent Meeting Texas State Medical Association.... 502
The Medical College Question.............................. 389
Tall Oaks from Little Acorns............................... 334
The Work of Texas Surgeons................................ 415
Urethra, Rupture of ........................................ 31a
Unique Case in Minor Surgery, a........................... 197
Uterine Fibro-Myoma, complicating labor................ 447
" Febroid, an immense.................................... 402
Unique Case, a............................................ 5, 402
Urethra, Congenital Stricture of........................... 355
Veratrum in Pueperal Eclampsia ........................... 228
Van Zandt Co. Medical Association.....................418, 377
Vick's Floral Guide ........................................ 294
Vital Statistics ............................................ 390
Works of Hypocrates, the genuine......................... 84
What are we here for ? ..................................... 29
Welch, Dr. Albert, Death of................................ 173
Water Melon Seed in the Trachea, a ...................... 66
West Texas Medical Association..........................281, 240
What are the Duties of a Publishing Committee.......... 423

